# Understanding the Multiple Influences on Black Parents’ School Involvement: A Longitudinal Perspective

**DOI:** 10.3390/children11060722

**Published:** 2024-06-13

**Authors:** Adrian Gale, Ed-Dee Williams, Donte Boyd, Husain Lateef

**Affiliations:** 1 School of Social Work, Rutgers University, New Brunswick, NJ 08901, USA; 2 School of Social Work, Boston College, Chestnut Hill, MA 02467, USA; willibzu@bc.edu; 3 College of Social Work, The Ohio State University, Columbus, OH 43210, USA; boyd.465@osu.edu; 4 Brown School, Washington University in St. Louis, St. Louis, MO 63130, USA

**Keywords:** parenting, Black parents, grades, parent involvement, parent efficacy for involvement, parent involvement attitudes

## Abstract

This study explores longitudinal influences of various factors on Black parents’ involvement in their children’s education. Guided by Hoover-Dempsey & Sandler’s Model of Parent Involvement, this research examines whether parents’ school climate perceptions, attitudes about involvement, self-efficacy, and children’s academic performance predict parent involvement over time. Utilizing data from the Maryland Adolescence in Context Study with a sample of 560 Black parents, we found that positive school climate perceptions and favorable attitudes towards involvement significantly predict increased parent involvement in later years. The results underscore the importance of supportive school environments and parent attitudes in fostering their involvement.

## 1. Introduction

Parents’ involvement in their children’s schooling (e.g., joining school activities and helping with homework) significantly increases their children’s chances for academic success [1,2,3]. For example, children whose parents are more involved tend to have higher grades, better test scores, fewer absences, and higher high school graduation rates [4,5]. Yet, there is still much to learn about specific factors that lead parents to be involved in their children’s schooling. Previous research has found that sociodemographic factors such as family income and education, parents’ marital status, and number of children are all antecedents of parent involvement [6,7,8]. Among researchers and policymakers, there is concern that Black parents are less involved in their children’s schooling than parents of other races [9,10]. Given the many benefits of parent involvement in their children’s outcomes, understanding factors influencing involvement may increase Black parents’ involvement. Therefore, this paper examines several factors that influence parent involvement in homework. These factors are parents’ perceptions of the climate at their children’s school, their attitudes towards involvement, their feelings of efficacy and prior involvement, and students’ grades.

## 2. Theoretical Framework

Our conceptualization of the antecedents of parent involvement is guided by Hoover-Dempsey and Sandler’s Model of Parent Involvement [11,12,13]. According to this model, various ecological factors (individual and environmental) influence parent involvement in their children’s education. Researchers have found that antecedents of parent involvement fit into three related categories. First, parents may become more involved if they perceive being an involved parent as an important role for parents to play [14]. Second, parents may become more involved if they believe they have the skills to effectively help their children succeed in school [15]. Finally, parents may become more involved when they view their children’s school environment as welcoming [16,17]. Parents are involved in their children’s schools (e.g., attending parent-teacher meetings; [18]) or at home (e.g., helping with homework; [19,20]). The current study employs Hoover-Dempsey and Sandler’s Parent Involvement Model because it is relevant to parents in the 21st century who continue to make involvement decisions.

Parent involvement decisions may be particularly crucial for Black parents [16]. Prior studies have found that Black parents may harbor negative feelings toward schools because overt racism or discrimination may cause Black parents to take a hostile stance when interacting with their children’s schools [21]. Further, even when they receive an invitation to a school event, Black parents may be hesitant to become involved if they believe that school officials have biases and discriminatory attitudes toward them [22,23]. Therefore, learning about the factors that impact Black parents’ involvement is important. We test Hoover-Dempsey and Sandler’s Parent Involvement Model by examining specific antecedents of parent involvement among Black parents.

### 2.1. Perceptions of School Climate

Parents’ perceptions of the school climate play a role in parents’ behavior and beliefs [16]. School climate refers to the school norms as they are perceived and interpreted by individuals [24,25]. Thus, parents’ school climate perceptions refer to parents’ perceptions about the happenings in their children’s schools. Previous studies have shown that parents’ school climate perceptions influence their beliefs and behaviors [16]. When parents perceive their children’s schools as welcoming places where their children are given the best chance to succeed, they are more likely to be involved. For instance, recent research by [26] found that school invitations, a correlation of parents’ school climate perceptions, were related to higher parent involvement. Notably, this association was stronger for the Latino sample than for the Black or non-Latino sample. Similarly, [17] found that parents with more positive perceptions of the climate in their children’s schools (i.e., parents felt welcomed and helped by school staff) were more likely to be involved in their children’s school (e.g., volunteer in school activities and attend parent-teacher meetings). Concerning Black parents’ involvement, the sample for [17] study only had 17.4% Black parents, who reported less involvement than the rest.

The literature on school climate perceptions suggests significant associations with parent involvement. However, little research has examined the link between school climate and involvement among Black parents. Because Black parents are likely to feel less welcome in their children’s schools than parents of other races [27], it is still unclear whether school climate perceptions are associated with parent involvement among these parents. Though aged, the sample for the current study represents the largest sample of only Black parents to examine the link between parents’ school climate perceptions and parent involvement.

### 2.2. Parents’ Attitudes about Involvement

Parents’ attitudes about involvement are also related to parents’ actual involvement. Parents’ attitudes about involvement refer to their beliefs about the extent to which they should be involved in their children’s education [28,29,30]. The literature on parent attitudes suggests that parents who view themselves as essential agents in their children’s education are more likely to be involved in educational activities in general. Parents who believed that their children’s education was solely the job of the schools were less likely to be involved [18,31]. Parents’ attitudes about involvement may also be connected to their cultural and ethnic backgrounds [32]. For instance, ref. [32] investigated parent involvement attitudes in a sample of White and Asian American parents. They found that Asian American parents reported stronger attitudes about involvement in learning academic skills than European Americans. However, European American parents had stronger attitudes about involvement in school events than Asian American parents. Despite these findings about cultural influences on parents’ involvement attitudes, little is known about the link between Black parents’ attitudes about involvement and their involvement with their children’s homework.

### 2.3. Parents’ Efficacy in Helping Children in School

Parents’ efficacy in helping children succeed in school refers to the extent to which parents believe they have the skills and knowledge necessary to help with homework [12,33]. According to [12], self-efficacy comes from direct experience, vicarious experience, verbal persuasion, and emotional arousal. Research findings on parents’ self-efficacy in parent involvement have been somewhat mixed. That is, while some prior research has shown that parents’ self-efficacy is related to more parent involvement [12,34], other research has shown that higher levels of self-efficacy are related to lower parent involvement [35]. [36] found that African American fathers’ self-efficacy ratings were associated with greater involvement in education at home. On the other hand, [35] found that parents with higher self-efficacy were less likely to be involved among a sample of Latino parents. More research is needed to clarify the nature of the link between parents’ self-efficacy for helping children and parent involvement.

## 3. Present Study

The current study extends Hoover-Dempsey and Sandler’s 1997 parent involvement model by examining parents’ homework involvement antecedents in a sample of Black parents. In particular, this study examined longitudinal associations between student grades, parents’ school perceptions, and parents’ attitudes and feelings of efficacy about their ability to help their children with homework on parent involvement with their children’s homework. Previous research has demonstrated that parents’ beliefs about being welcome in their children’s school impacted their school involvement beliefs [17,26]. Thus, we hypothesize that more positive school climate perceptions will be associated with more involvement from parents.

We also investigate whether children’s grades were associated with their parents’ school involvement. Prior research has shown a positive association between children’s grades and parent involvement [37]. We hypothesize that children with higher grade point averages will have more involved parents. Lastly, we examine whether parents’ attitudes and feelings of efficacy about their ability to help their children with homework are associated with parent involvement. While past studies have generally found that parents’ attitudes and efficacy regarding involvement are antecedents to parent involvement [12,32,34], given the unique school experiences of Black parents, it is unclear whether attitudes and efficacy are related to parent involvement among Black parents. We hypothesize that parents with more positive attitudes about parent involvement and parents who feel the efficacy of helping their children with homework will be more involved.

## 4. Methods

### 4.1. Participants

This paper utilized two waves of survey data from the Maryland Adolescence in Context Study (MADICS), a community-based longitudinal study of junior high students and their families based in Prince George’s (PG) County, a suburb of Maryland. The first wave of data was collected in 1991, and the second wave was collected in 1995. The MADICS dataset has unique advantages for studying these research questions. PG County was selected because of its predominantly Black school population and the heterogeneity in the household socioeconomic status of Black families in this community [16]. In particular, the demographic makeup of the MADICS allowed us to examine parent involvement among a diverse sample of Black parents and children. The MADICS aimed to investigate the influence of social contexts on adolescent development.

The MADICS sample included 879 Black parents who first completed the survey at wave 1 when their children were in seventh grade, and 580 completed the survey four years later at wave 4 when their children were in eleventh grade. The significant loss of participants (36%) was primarily related to dropout at wave 4. Little’s chi-square test for missing data was significant (*ꭓ*^2^(44) = 55.887, *p* = 0.108), meaning we can assume that data were completely missing at random. Consequently, listwise deletion of cases with missing values will likely not bias results. After accounting for and removing participants through listwise deletion, the final sample of Black parents used for analyses was 560. The final sample did not differ significantly from the missing data on school climate perceptions, parent attitudes regarding parent involvement, family income, or gender. However, among those excluded from analyses, parents had lower levels of education and efficacy regarding parent involvement, while children had lower GPAs in seventh grade. The mean age of the 560 participants included in the analyses in seventh grade was 12.25 years (range of 11–14 years), and in eleventh grade, the mean age of participants was 16.28 (range of 15–18 years). The male-to-female ratio in the adolescent sample was approximately equal (51% male to 49% female).

### 4.2. Measures

*Parent involvement (Eleventh grade)*. Youth reported on their parents’ level of involvement in their children’s schools using a three-item scale. Parents responded to the question: How often do the following things happen? A sample item for this scale includes the following: “Your parent(s) helps you with your homework after it’s completed; for example, checking that it’s done correctly or proof-reading reports during the school year.” Parents responded using a 6-point Likert scale from one (*almost never*) to six (*almost every day*). Higher scores on this scale indicated greater parent involvement (α = 0.71).

*Parents’ school climate perceptions (Seventh grade)*. Parents’ perceptions of school climate were measured using a five-item scale, which asked about parent perceptions of the availability and receptiveness of school staff. Parents responded to five prompts about the climate of their child’s school. Parents answered using a 5-point Likert from (one (*strongly disagree*) to five (*strongly agree*)). A sample item for this scale is “*Children generally feel that they belong*”. Higher scores on this scale indicated more positive perceptions of the school climate (α = 0.84).

*Seventh-grade academic achievement.* Academic achievement was measured using the target youth’s seventh-grade GPA from school records. GPAs were measured on a scale from 1 (*F*) to 5 (*A*).

*Parent involvement attitudes (Seventh grade)*. The MADICS research team developed the parent involvement attitudes scale. This 8-item scale asked about parents’ attitudes about becoming involved in their children’s schools. The response scale ranged from (*1 = not at all to 4 = a lot*). One sample question included the following: “I am not interested in doing things at school.” Higher scores on this scale indicated more positive attitudes about being involved in their children’s schools (α = 0.68).

*Parent self-efficacy for homework help (Seventh grade)*. The MADICS research team developed the parent efficacy scale. This 3-item scale asked about parents’ beliefs that they can help their children with their homework. The response scale ranged from (*1 = not at all to 4 = a lot*). “How much can you do to get your 7th grader to do (his/her) homework?” Higher scores on this scale indicated increased parent efficacy in helping their children with homework (α = 0.79).

*Demographic measures and statistical controls*. The sociodemographic characteristics of the target adolescents and their families were used as covariates. These measures included parent-reported total pre-tax family income in 1990 on a scale from 1 (*less than $5000*) to 16 (*more than $75,000*), with each number representing a range of USD 5000 and parents-reported highest level of education, which was recorded into three categories to represent did not graduate high school, finished high school/received a GED and graduated from college/had at least a college degree. Finally, children self-reported their gender.

## 5. Data Analysis Plan

The assumptions for the analyses were tested using SPSS. Multivariate normality was evident in quantitative analysis as both skewness and kurtosis did not differ significantly from zero. The current study examined data from the same sample at two time points four years apart. We utilized a predictive model over time with seventh-grade dependent variables predicting parents’ involvement in the eleventh grade. One ordinary least squares regression model was used to answer the main study questions. Parent involvement in eleventh grade was the independent variable in this regression, and perceptions of school climate, GPA, parents’ involvement attitudes, and parents’ self-efficacy were the dependent variables. Additionally, previous studies have shown that income, parents’ educational level, and children’s gender are associated with parent involvement [8,37]. Thus, these variables were used as statistical controls in the regression.

## 6. Results

### 6.1. Descriptives and Correlations

Means, standard deviations, and intercorrelations are presented in Table 1. Our analyses used Pearson correlations to reveal small and positive correlations between parent involvement and school climate perceptions, involvement attitudes, and self-efficacy.

### 6.2. The Association between Independent Variables and Parent Involvement

The omnibus test for the analytic model (Table 2) was significant (F (7, 472) = 5.957, *p* < 0.001); adjusted R^2^ = 0.08. Concerning the covariates, parents’ education level was associated with parents’ involvement in eleventh grade (β = 0.129, *p* < 0.05). On the other hand, neither family income (β = 0.047, *p* = 0.354) nor children’s gender (β = 0.036, *p* = 0.431) were associated with parents’ involvement while their children were in eleventh grade. With respect to the independent variables of interest, parents’ school climate in seventh grade (β = 0.142, *p* < 0.01) was positively associated with parent involvement in eleventh grade. However, there was no significant association between grade point average in seventh grade and parent involvement in eleventh grade (β = −0.018, *p* = 0.735). Parent involvement attitudes were positively associated with parent involvement in eleventh grade (β = 0.12, *p* < 0.05). Lastly, parents’ involvement self-efficacy was not associated with parent involvement in eleventh grade (β = 0.040, *p* = 0.424).

## 7. Discussion

Guided by Hoover-Dempsey and Sandler’s Model of Parent Involvement [11], this paper conducted a longitudinal analysis to examine antecedents to parents’ involvement in their children’s homework. These data were collected from a sample of Black parents over two waves (when their children were in seventh and again in eleventh grade). In line with much of the prior scholarship on antecedents to parent involvement, parents’ school climate perceptions and parents’ attitudes about involvement for helping with homework all predicted parent involvement in eleventh grade. These findings highlight the multiple influences on Black parents’ involvement in their children’s involvement. Our examination of these factors as antecedents to involvement in a sample of Black parents expands the literature on parent involvement.

### 7.1. Parents’ School Climate Perceptions and Parent Involvement

In keeping with prior research [26], parents’ perceptions about the climate at their children’s school were associated with parent involvement. Notably, this finding remained statistically significant even after controlling for the effects of children’s gender, family income, and parents’ education. This finding has implications for parents as well as for schools. Specifically, these findings underscore the critical role of parents’ relationships with schools in their parenting behaviors. Furthermore, our results indicate that parents’ school climate perceptions impacted their involvement in homework. Thus, parents’ feelings about their children’s schools appear to influence involvement outside school (i.e., homework). Given the link between parents’ school climate perceptions and their involvement, we believe that efforts should be made to cultivate positive relationships between Black parents and their children’s schools.

### 7.2. Parent Involvement and Students’ Achievement

Contrary to our hypotheses, children’s seventh-grade GPA was not associated with their parent’s involvement in eleventh grade. This contradicts previous research findings showing a link between academic performance and parent involvement [38]. Specifically, ref. [38] found that children with higher academic performance in fifth grade reported more homework help from their parents in seventh grade. The current findings suggest that parents in our sample did not consider their children’s seventh-grade GPAs when making involvement decisions in eleventh grade. It could be that more recent grades (within one or two years) could be more influential on parent involvement. One reason for the current findings may be that, as shown in previous research, parent involvement declines as children advance through school [37]. Thus, the four years that separated observations may have diluted the connection between GPA and parents’ involvement. Future studies should examine the circumstances under which children’s grades and other academic performance metrics may influence parent involvement.

### 7.3. Parent Involvement and Parent Involvement Attitudes

To our knowledge, this study is among the first to explicitly assess the association between parent involvement attitudes and parents’ involvement attitudes. Consistent with our hypotheses, parent involvement attitudes when their children were in seventh grade were positively associated with parent involvement in homework during their children’s eleventh-grade year. This finding provides further evidence that parents’ beliefs or attitudes influence parenting behaviors (i.e., involvement). Among Black parents, our results suggest that having a positive attitude towards involvement is a precursor to their greater involvement in their children’s homework. While our finding regarding parent involvement attitudes helps increase knowledge about beliefs that may lead Black parents to become involved, there is still a dearth of literature regarding how Black parents’ involvement attitudes are formed. More research should investigate the factors that impact parent involvement attitudes (e.g., cultural beliefs; [32]) as this may provide more insight into influences on parent involvement.

### 7.4. Parent Involvement and Parents’ Self-Efficacy in Helping Children

The current study is among the first to examine the association between parents’ self-efficacy and involvement over time (four years). It is also among the first to investigate this association in a sample of Black parents. Interestingly, parents’ self-efficacy for helping children with homework for their seventh-grade children was not associated with parent involvement in eleventh grade. This finding aligned with past research demonstrating that parents’ self-efficacy did not predict parent involvement [35]. It could be that parents’ self-efficacy may not be sufficient to inspire their involvement. For instance, ref. [35] found that parents’ self-efficacy predictive of parents’ involvement was only in combination with perceived teacher invitations. More research is needed to investigate the circumstances under which self-efficacy leads to more involvement.

### 7.5. Limitations and Future Directions

While the current findings contribute to scholarship on parent involvement among Black parents, it is important to acknowledge certain limitations and suggest areas for further research. First, our measure of parent involvement was only two questions about homework. A more robust measure of parents’ homework help may better detect differences. Further, only children reported their parents’ involvement in homework help; it is unclear whether this is an accurate estimate of parents’ involvement. Therefore, future research should examine antecedents to parents’ reports of their involvement in their children’s schooling.

Another limitation of this study is the age of the dataset, which includes data from 1991 (seventh grade) and 1995 (eleventh grade). While we argue that parents’ participation in education has not fundamentally changed, it is crucial to acknowledge that much has evolved in the past three decades, especially regarding technology and internet use in schooling. Advances in technology have introduced new ways for parents to engage in their children’s education. Yet, the messaging around Black parents’ involvement in their children’s education remains negative [9]. Current perceptions of school climate may now be influenced by parents’ online interactions with teachers and schools.

Furthermore, generational differences between parents from 1991–1995 and those in 2024 could impact the results. For example, parents in the early ‘90s might have emphasized traditional forms of involvement, such as attending parent-teacher conferences and helping with homework. In contrast, parents today may prioritize digital communication with teachers and support their children’s learning through online resources and educational apps [39]. These shifts in values and habits could influence parents’ involvement differently. Therefore, it would be worthwhile to reference generational characteristics in the literature and research descriptions, as they greatly affect the interpretability and usefulness of the results.

Despite these considerations, this study’s mean levels of parent involvement align with more recent empirical studies (e.g., [10]). Additionally, we maintain that there is no reason to believe that Parent Development Theory would operate differently for parents in 1995 compared to those in 2024. Future research should continue examining the antecedents of parent involvement among Black parents, considering technological advancements and generational shifts.

### 7.6. Implications

There is a consensus that parents play a central role in educational experiences, academic achievement, and success factors associated with the positive effects of educational success. However, prior scholarship has identified that parent participation may be lower among Black Americans than parents from other ethnic and racial populations [40]. Subsequently, and in light of evident structural barriers and social inequities that may contribute to lower levels of parent involvement among Black parents [41], our paper makes significant contributions toward identifying potentially sustainable implications factors for practice that may bolster the parent involvement of Black parents in schools.

First, our findings that among Black parents’ school climate perceptions (how they perceive their child’s schools as welcoming towards them and their child) was related to their involvement supports the mezzo level scholarship on the importance of institutions towards improving individuals’ prosocial behaviors [42]. Practically, this translates into the importance of schools and related administrative units of schools to prioritize evaluations of district and individual school climate perceptions of parents. Disaggregating these data by race and ethnicity may inform equitable directions to improve such perceptions for all students and their families. Moreover, the school climate finding adds important nuance to the potential weight of more micro-level individual factors instead of micro-level home factors (e.g., household income, parents’ marital status) that is sorely needed in research on Black Americans [43]. Indeed, our significant school climate-parent involvement finding contrasts with our finding that another micro-level factor (i.e., seventh-grade GPA) did not contribute to Black parent involvement. Schools should be conscious not to bias perceptions of parents’ involvement in students’ achievement and instead prioritize ways to improve the relationship between the school and the parents to increase participation.

Another significant implication of our findings is that enhancing parents’ belief in their contributions to their children’s education may be crucial for increasing their involvement in homework [44]. Prior scholarship supports that while there is a heterogeneity of experiences of Black familial households, for some Black families, particularly those in urban communities, many face a cluster of barriers that may strain capacities to feel empowered that their contributions are substantially impactful for their child’s success in schools. Subsequently, schools can play a crucial role, providing parent literacy content that demonstrates their importance and providing potential community-based workshops or support mechanisms to improve parents’ self-efficacy for Black parents’ part in their child’s life.

## 8. Conclusions

National efforts to increase parent involvement are one of the main thrusts of many efforts by teachers and parents [13]. These efforts to increase parent involvement should consider Black parents’ perceptions about their children’s school, their attitudes about involvement, and their involvement efficacy as possible avenues to increase their involvement. Consistent with Hoover-Dempsey and Sandler’s Model of Parent Involvement [11], our findings highlight that Black parents’ beliefs about their children’s schools, involvement attitudes, and self-efficacy for involvement are important. Schools should seek to ensure that parents feel that schools are welcoming to Black parents. By providing a welcoming environment for parents and supporting attitudes and feelings of self-efficacy, schools may increase parents’ involvement and, by extension, improve student achievement.

## Figures and Tables

**Table 1:** Correlations and descriptives for key study variables.

	1	2	3	4	5
1. Perceptions of school climate (7th grade)	-				
2. GPA (7th grade)	0.119 **	-			
3. Parents’ Involvement Attitudes (7th grade)	−0.102 **	−0.174 **	-		
4. Parents’ Self Efficacy (7th grade)	0.176 **	0.325 **	−0.260 **	-	
5. Parent Involvement (11th grade)	0.161 **	0.083	−0.152 **	0.149 **	-
Mean	3.67	3.42	1.33	3.68	3.17
S.D.	0.58	0.87	0.43	0.44	1.08

** *p* < 0.01.

**Table 2:** Summary of regression analyses for antecedents of parent involvement (11th grade).

Variable	*b*	SE	β
Parents’ Education Level	0.059	0.023	0.129 *
Family Income	0.012	0.013	0.047
Child gender	0.077	0.098	0.036
Perceptions of school climate (7th grade)	0.260	0.083	0.142 **
GPA (7th grade)	−0.021	0.062	−0.018
Parents’ Involvement Attitudes (7th grade)	−0.321	0.126	−0.120 *
Parents’ Self Efficacy (7th grade)	0.101	0.126	0.040
R^2^		0.081	
F for change in R^2^		5.957 ***	

* *p* < 0.05. ** *p* < 0.01. *** *p* < 0.001.

## Data Availability

The data used in the current study are available to the public. These data can be accessed here: https://garp.education.uci.edu/madics.html.
